# Burden of Congenital CMV Infection: A Narrative Review and Implications for Public Health Interventions

**DOI:** 10.3390/v16081311

**Published:** 2024-08-17

**Authors:** Cecilia Liberati, Giulia Sturniolo, Giulia Brigadoi, Silvia Cavinato, Silvia Visentin, Erich Cosmi, Daniele Donà, Osvalda Rampon

**Affiliations:** 1 Department of Women’s and Children’s Health, Pediatric Infectious Disease, Padua University Hospital, 35126 Padua, Italy; cecilia.liberati@unipd.it (C.L.); giulia.sturniolo@aopd.veneto.it (G.S.); daniele.dona@unipd.it (D.D.); osvalda.rampon@aopd.veneto.it (O.R.); 2 Infectious and Tropical Diseases Unit, Padua University Hospital, 35126 Padua, Italy; silvia.cavinato@aopd.veneto.it; 3 Department of Women’s and Children’s Health, Gynecological and Obstetric Clinic, Padua University Hospital, 35126 Padua, Italy; silvia.visentin1@unipd.it (S.V.); erich.cosmi@unipd.it (E.C.)

**Keywords:** neonatal screening, maternal screening, cytomegalovirus, CMV vaccines, CMV awareness

## Abstract

Cytomegalovirus causes the most common congenital infection worldwide. With most infants asymptomatic at birth, the few affected may present with variable clinical scenarios, from isolated hearing loss to severe neurologic impairment. Public health interventions include all actions at the health system, community, and individual levels that aim at reducing the burden of congenital Cytomegalovirus. This review examines the literature on maternal and neonatal screening programs in light of current evidence for treatment and the development of vaccines against Cytomegalovirus. Potential biases and benefits of these interventions are outlined, with the objective of increasing awareness about the problem and providing readers with data and critical tools to participate in this ongoing debate.

## 1. Introduction

The burden of congenital CMV infection (cCMV) is related to the sequelae of fetal invasion, possibly leading to irreversible complications. In screened populations, 40 up to 58% of symptomatic and 13.5% of asymptomatic infants at birth develop permanent sequelae [1]. Neurologic sequelae, including sensorineural hearing loss (SNHL), are mostly limited to the acquisition of primary infection during the first trimester of pregnancy [2,3,4], and symptomatic newborns seem at higher risk for developing delayed-onset SNHL [5]. Although treatment for cCMV has been used for more than 20 years, evidence is still weak, with data coming from a few clinical trials (from the Collaborative Antiviral Study Group—CASG) and observational studies [6,7,8,9]. Based on current evidence, treatment is considered for symptomatic infected children with severe disease or with at least one organ involved [10]. In Europe, no treatment is indicated in asymptomatic children or mild disease cases [11]. In fact, all treatment studies excluded asymptomatic children, so no recommendation is available for this category. Given the lack of evidence, some European experts suggest treating isolated SNHL cases. In the same way, weak data denote antiviral utilization for delayed-onset SNHL [12,13], with some ongoing trials [14,15]. Regarding the treatment of pregnant mothers, no evidence strongly indicates the need for antiviral therapy. However, valacyclovir therapy of periconceptional or first-trimester primary CMV infection seems to reduce vertical transmission and neonatal infections [16]. Hyperimmune CMV globulin seem to have a role in decreasing the inflammatory response to CMV and subsequent tissue damage [17].

**Figure 1:**
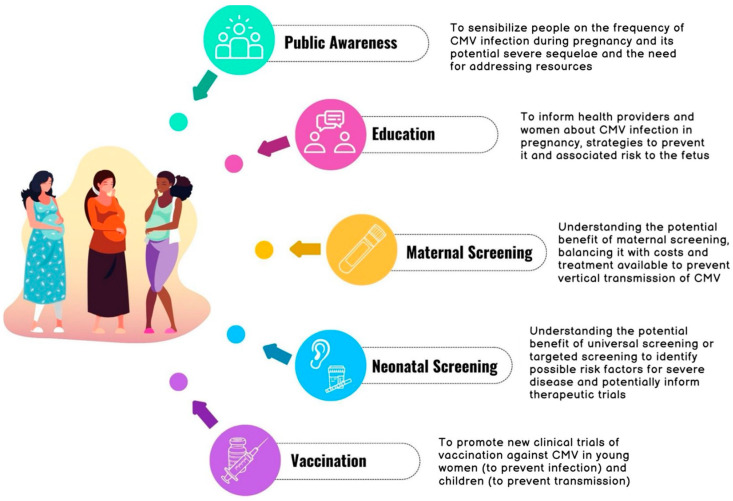
Preventive public measures proposed to reduce the cCMV burden. Adapted from Public Health Interventions (population-based), Minnesota Department of Health [18].

Being the most widespread congenital infection, several public health interventions aiming at reducing the burden of disease have been proposed (or rejected) by health systems and public authorities, leaving space for wide debates still without a clear answer. Public intervention should include the epidemiology and surveillance of the disease, outreach to the population of interest (pregnant women), screening programs, health teaching, vaccination plans, and policy development (Figure 1). The aim of this review is to critically appraise public health interventions reported in the existing literature to reduce the burden of cCMV, with a special focus on neonatal screening studies and vaccine development.

## 2. Materials and Methods

To explore cCMV public awareness strategies, a literature search on PubMed was conducted between January 2000 and December 2023 and on national and international society websites in 2024. This search was restricted to original English-language studies and websites.

Concerning neonatal screening, studies focusing solely on epidemiological aspects or on the performance of different diagnostic methods were excluded. Additionally, studies where the targeted population was identified based only on maternal history or neonatal clinical suspicion of infection were excluded. It is reasonable to assume that these infants would be tested regardless and thus would not benefit from a screening program.

Vaccine studies for CMV were included if they involved the population of interest (adolescents, children, and women of childbearing age). Vaccine studies on transplant recipients and phase 1 trials were excluded.

## 3. Results

### 3.1. Awareness and Education in Pregnancy

The greatest risk to pregnant women is exposure to the urine or saliva of young children. Information on CMV disease in pregnancy would properly engage the women in prevention strategies, strengthening hygienic precautions that will reduce the possibility of CMV infections among others [19,20,21,22]. Physicians and obstetricians should raise awareness and provide education, these actions still being largely unpracticed [23]. A systematic review by Barber et al. on the pregnancy prevention of CMV acquisition through hygiene-based behavioral interventions showed that preventive and hygienic measures are acceptable to pregnant women and have the potential to reduce the risk of infection during pregnancy [24]. In the latest update of the National Institute for Health and Care Excellence (NICE) guidelines for antenatal care, it is recommended that at the first antenatal appointment, women are provided with information on infections that can impact pregnancy, including CMV, and that their personal risk factors and concerns (such as occupation and living with other children) are discussed [25].

The Center for Disease Control and Prevention (CDC) offers a dedicated website providing information to parents about the disease and its prevention, with fact sheets and an infographic [26]. CDC since 2020 is promoting a CMV Awareness Month to increase awareness.

In the UK, the “CMV Action” organization promotes knowledge, supports families, and promotes scientific advances in the field of congenital CMV [27]. Such great initiatives are still rare and are of paramount importance in terms of general awareness.

### 3.2. Maternal Screening

The identification of CMV infection during pregnancy can cause significant maternal anxiety because of the uncertainty of what the significance of the disease will be and leads to more testing and the possible (unnecessary) termination of pregnancy [28]. Practices about maternal screening are heterogeneous in the absence of an international consensus and recommendations [29]. Several considerations need to be carried out when balancing the feasibility of maternal serologic screening. First of all, almost half of congenital infections occur as non-primary maternal infection with CMV rather than primary infection, even if the risk of vertical transmission is lower in this case (<3.5% in CMV-seropositive mothers versus 32% during primary infection [30]). Reinfection is, however, the main modality of acquiring cCMV in high-seroprevalence populations, which are mostly low–middle income countries [31]. In the United States of America, only 25% of infants with cCMV were attributable to a primary maternal infection, with three quarters of cases born to immune mothers [32]. Secondly, invasive testing procedures (amniotic fluid PCR and viral culture combined) yield almost 100% positive and negative predictive values for congenital infection [20], but an association between viral loads and symptomatic infection at birth is debated, with no clear evidence [33]. If reinfection is at a lower risk for mother-to-child transmission, infants born to immune mothers are still at risk of symptomatic infection and SNHL. This was shown in a Brazilian prospective study (with a 96.7% CMV-immune population rate), where 60% of SNHL affected infants were born to a mother with non-primary CMV infection [34]. Maternal serologic screening alone therefore misses a considerable part of vertical infections and affected children. With these uncertainties, guidelines until now have suggested against the universal screening of CMV during pregnancy [29]. However, the recently published consensus from the European Congenital Cytomegalovirus Initiative made important recommendations on treatment options for pregnant women with primary CMV infection, suggesting valacyclovir therapy and, in some instances, considering CMV hyperimmune globulin. These recommendations are based on available studies. Two randomized controlled trials on the use of CMV hyperimmune globulin during pregnancy did not show a reduction in the vertical transmission of the infection or clinical outcomes at birth [35,36]. However, in a trial carried out by Revello, a reduction in vertical transmission from 40% to 30% was observed, even if not reaching significance because of the limited sample size. On the other hand, the utilization of hyperimmune globulin was associated with an increased incidence of obstetrical adverse effects [35]. Some earlier data suggest the potential role of CMV hyperimmune globulin in reducing the risk of poor clinical outcomes during infant follow-up [37,38,39]. In the same way, a retrospective database study showed that high-dose hyperimmune globulin administration was associated with a lower rate of fetal infection [40]. In recent years, studies on valacyclovir have shown promising results in reducing the rate of vertical transmission [16,41,42,43]. Other antivirals have shown ex vivo efficacy in first-trimester placental trophoblast cell cultures and third-trimester placental explant histocultures [44]. With these treatment options, maternal screening in the first trimester becomes crucial to identify women who would most likely benefit from treatments and to enhance hygiene measures and education for seronegative women [17].

### 3.3. Neonatal Screening

The screening of neonates would identify all children suffering from this condition. Available methods (PCR on saliva and urine) are, in fact, highly sensitive and specific. Universal screening for CMV at birth is not currently included in legislation in most countries. A debate among experts was raised in recent decades due to the potential benefit of this action (diagnosis, treatment, and follow-up), which was counter-balanced by the fact that most infections are asymptomatic and evidence for treatment does not demonstrate a strong benefit [9].

Many countries, regarding law indications or internal local practice, are adopting targeted CMV screening for infants failing the neonatal hearing screening (NHS). Currently, many countries adopt NHS [45] through evoked otoacoustic emissions (OAEs) or an automated measurement of auditory brainstem responses (ABRs), recommended for all infants before the age of three months in the United States, the United Kingdom, and Europe [46].

#### 3.3.1. Universal Screening

The studies included are summarized in Table 1.

Universal screening would diagnose asymptomatic babies, including isolated SNHL or asymptomatic abnormal brain findings. In the most numerous series from Fowler (USA), 8% of cCMV cases were found with isolated SNHL [47]. Other smaller studies obtained variable percentages, from 0 to 10%. In the study by Lorenzoni et al., universal screening was performed on premature and small-for-gestational-age newborns, revealing an increased incidence of cCMV and isolated SNHL (17%) [48]. The treatment and clinical outcomes of these children are largely unreported in the series analyzed: only four studies reported treatment and short-term outcomes. For this reason, coupled with the uncertainty of valganciclovir efficacy in asymptomatic infants, the long-term clinical significance of universal screening is hard to estimate. Larger, prospective, and longer studies of coupled universal screening with therapeutic trials would further clarify the benefit.

**Table 1:** Summary of studies included on universal screening for congenital CMV (cCMV). SNHL: sensorineural hearing loss; PCR: polymerase chain reaction; LO-SNHL: late-onset SNHL; SGA: small gestational age; OAEs: otoacoustic emissions; ABRs: automated measurement of auditory brain-stem responses; FP: false positive; DBS: dried blood spot; CNS: central nervous system; HL: hearing loss; US: ultrasound; gw: gestational week; n.a.: not applicable.

Author, Year, Ref, Country	Study Design and Time of Enrollment	Population	Method of Universal Screening	Results	Symptomatic Newborns at Birth (Other than Isolated HL)	Isolated HL Confirmed Cases	Treatment	Outcome	Comments
Schlesinger, 2005 [49] Israel	Multicenter prospective study From May 98 to August 99	Live-born infants	PCR on urine	14 diagnosed/2000 screened for CMV	2 symptomatic (microcephaly, hepatitis)	no HL found	n.a.	n.a.	This study did not identify any isolated SNHL, no information about follow-up for LO-SNHL was provided.
Lorenzoni, 2013 [48] Italy	Monocenter prospective From January 2012 to July 2013	Premature newborns (<37 gw) and SGA term infants (weight <3rd percentile)	PCR on urine	12 (10 preterms, 2 SGA)/383 screened/504 premature or SGA	1 preterm (lissencephaly)	2	n.a.	n.a.	Increased incidence of cCMV and isolated SHL (17%) in this populations.
Barkai, 2014 [50] Israel	Single-center prospective study From May 2011 to May 2012	Live-born infants	PCR on saliva confirmed by urine	48 cCMV/9845 screened for CMV/10,137 live-born infants	0	1	4 infants	1 LO-SNHL at 3 months of age	Incidence of neonatal hearing loss: 2%. The infant diagnosed with HL passed the OAE screening and was confirmed on ABR.
Fowler, 2017 [47] USA	Multicenter prospective From March 2007 to March 2012	Live-born infants	PCR on saliva or DBS	443 cCMV identified out of 100,332 tested	n.a.	35 confirmed	n.a.	n.a.	Incidence of neonatal hearing loss: 8%. The lack of CMV confirmation on urine may give some FP patients. 15 cCMV cases with confirmed SNHL passed the OAE screening.
Dar, 2017 [51] India	Multicenter, prospective study From December 2010 to May 2012	Live-born infants	PCR on saliva	20 diagnosed/1720 screened	1	2	n.a.	n.a.	Incidence of neonatal hearing loss: 10%. 1 out of 2 neonates with cCMV and SNHL passed the initial HS. The lack of CMV confirmation on urine may give some FP patients.
Yamamoto, 2020 [52] Brazil	Multicenter, prospective study From September 2013 to April 2017	Live-born infants	PCR on saliva, confirmed on urine	68 diagnosed/11,900 tested	4	4	7	Neonatal SNHL: between the 4 isolated SNHL, 1 progressed and 1 was stable at 18–48 months at follow-up For the 4 symptomatic babies, all had SNHL, one progressive and 3 stable at follow-up. For the other 49 cases, no late-onset HL was detected at a median 36-month follow-up	Incidence of neonatal hearing loss: 5.8%. 1 neonate with cCMV and SNHL passed the initial HS. Targeted screening would have missed 12.5% of infants with SNHL.
Yamada, 2020 [53] Japan	Multicenter prospective study From November 2009 to March 2018	Live-born infants	PCR on urine	56 diagnosed/11,736 tested for CMV	19	4	n.a.	Between the 4 isolated SNHL, 2 normal development and 2 mild sequele.	The incidence of isolated SNHL in this population is 7.1%.
Blazquez-Gamero, 2020 [54] Spain	Prospective, monocenter From February 2017 to February 2018	Live-born infants	PCR on saliva, confirmed by urine	15 positive out of 3190 tested	2	0	n.a.	No infants (13 available at follow-up) developed SNH at 25 months	The incidence of isolated SNHL in this population is 0%.
Letamendia-Richard, 2022 [55] France	Monocenter, retrospective From single unit, 2016–2020	Live-born infants	PCR on saliva at birth, confirmed by urine	63 confirmed infections/15,341 tested/15,649 live-born infants	8 infants small for gestational age, no one with HL	1	n.a.	n.a.	The child with isolated SNHL had hepatomegaly at prenatal US and his mother had known seroconversion, so it would have been diagnosed without intervention.
Chiereghin, 2022 [56] Italy	Multicenter prospective study From February 2019 to July 2020	Live-born infants	PCR on saliva confirmed by urine	21 confirmed cCMV/3151 screened for CMV	1 case with severe CNS disease and HL	1	2 (6 months)	1 asymptomatic infant developed LO-SNHL at 5 months of age	Incidence of neonatal hearing loss: 4.7%. No information regarding hearing screening test.

#### 3.3.2. Targeted Screening

The studies included are reported in Table 2.

Regarding targeted screening studies, they were mainly retrospective experiences from centers locally performing cCMV screening for infants failing NHS. The primary limitation in understanding the potential of screening is that most studies did not exclude symptomatic infants or those with known or suspected maternal infection during pregnancy. Even when considering isolated cases of sensorineural hearing loss (SNHL), these could still originate from mothers with known infections and be diagnosed without screening. Clinical outcomes are available from only two studies [57,58]. Furthermore, concerns exist regarding the sensitivity of hearing tests performed at birth. False negatives could result in missing cases of neonatal hearing loss, thereby depriving these children of the benefits of both pharmacological and non-pharmacological therapies and precluding early intervention for those with delayed-onset SNHL.

A prospective study by Fowler enrolled 443 children with cCMV identified through universal neonatal screening over 5 years: 35 newborns were diagnosed with SNHL by a confirmatory hearing test [47]. Of these 35 newborns, 15 (43%) passed their hearing screening. In this study, therefore, targeted screening missed 43% of cCMV with a neonatal-onset hearing deficit. One neonate out of two and one neonates out of four with confirmed SNHL passed the HS in the series from Dar (India) and Yamamoto (Brazil), respectively [51,52].

Another aspect that emerged during the search is that some infants classified as symptomatic within screening programs are, in practice, asymptomatic at birth. They are deemed symptomatic only due to laboratory abnormalities or abnormal cerebral findings that would otherwise remain undiagnosed [49,53,59].

In this context, it is worth questioning whether an asymptomatic newborn is truly ‘asymptomatic’. The significance of nonspecific ultrasound or MRI findings, such as isolated lenticulostriatal vasculopathy, remains uncertain, and the long-term outcomes associated with these findings are still unknown [11]. The follow-up of diagnosed cases would lead to the development of a scoring system aimed at more accurately quantifying the risk of adverse outcomes. This would expand the knowledge base to facilitate studies on treatment.

From the scant clinical data and follow-up availability in the search, it is hard to extrapolate a long-term clinical outcome of targeted and universal screening for cCMV. This would be clarified in the occurrence of clear treatment indications and evidence of efficacy. However, mainly due to the paucity of data and the fact that most cCMV cases are mild, large trials of treatment are missing, and recommendations are weak.

**Table 2:** Summary of studies included on targeted screening for congenital CMV. SNHL: sensorineural hearing loss; PCR: polymerase chain reaction; NHS: neonatal hearing screening; LO-SNHL: late-onset SNHL; OAE: otoacoustic emission; ABR: auditory brain-stem response; HIV: human immunodeficiency virus; HL: hearing loss; DBS: dried blood spot; CNS: central nervous system; n.a.: not applicable.

Author, Year, Ref, Country	Study Design and Time of Enrollment	Patient	Method of Targeted Screening	Results	Symptomatic Newborns at Birth (Other than Isolated HL)	Isolated HL Confirmed Cases	Treatment	Outcomes of cCMV Cases	Comments
Stehel, 2008 [60] Texas (USA)	Monocenter, retrospective From September 1999 to August 2004	Patients failing HS, mother infected with HIV, clinical or lab signs suggestive	PCR on urine	24 confirmed/483 screened/572 failing HS.	9	8	n.a.	n.a.	The inclusion criteria for screening were not stringent. It was hard to predict if the screening would be different to normal clinical practice.
Williams, 2014 [61] UK	Multicenter prospective From August 2010 to October 2012	Infants < 22 days old failing NHS. Known cCMV excluded	PCR on urine or saliva	6 diagnosed/407 screened/411 recruited after failing NHS	n.a.	3	n.a.	n.a.	Clinical data and outcome missing.
Kawada, 2015 [62] Japan	Prospective study From January 2011 to December 2013	Infants failing NHS	PCR on saliva or urine	6 confirmed out fo 127 failing NHS	0	6	valgancyclovir for 6 weeks	only 1 out of 6 improved at 1-year follow-up	Valganciclovir did not show to significantly improved hearing function.
Roth, 2017 [63] Israel	Single-center retrospective study From 2014 to 2015	Infants failing NHS	PCR on saliva confirmed by urine	4 confirmed cCMV/180 tested for CMV/200 failing NHS	2	3	n.a.	n.a.	Targeted screening identified 1 child (out of 200 failing NHS) who needed treatment. Outcomes missing.
Diener, 2017 [58] Utah (USA)	Retrospective multicenter From 2013 to 2015	Live-born infants failing NHS. Infants with suggestive symptoms were excluded	PCR on saliva, confirmed on urine	14 diagnosed/314 screened for CMV/509 failing HS	0	6	n.a.	n.a.	No information on follow-up and outcome.
Rawlinson, 2018 [59] Australia	Monocenter, retrospective study From October 2009 to Oct 2016	Infants failing HS and formal audiological testing (ABR)	PCR on saliva up to 2011, after 2011, positivity on saliva was confirmed on urine	19 diagnosed/323 screened/502 infants with confirmed HL	4	15	6 out of 19 (only 4 started within the first month of life)	n.a.	No clinical outcome, no follow-up. Symptomatic infants were not excluded from the study (4 out of 19 confirmed) and were reasonably diagnosed without this intervention.
Beswick, 2019 [64] Australia	Multicenter, retrospective From August 2014 to April 2016	Neonates failing NHS (twice OAE)	PCR on saliva, confirmed by urine and blood	3 diagnosed out of 234 screened/347 failing NHS	0	2	1, valganciclovir	n.a.	Intervention allowed diagnosis and treatment of one otherwise asymptomatic infant. No clinical outcome provided.
Pellegrinelli, 2019 [65] Italy	Observational single-center study From 2014 to 2018	Infants failing NHS (AOE)	PCR on DBS	5 DBS tested positive/82 DBS screened/89 failing NHS	n.a.	5	n.a.	n.a.	DBS method may have missed some CMV diagnoses.
Ronner, 2021 [57] Massachusetts (USA)	Monocenter, retrospective chart review, From 2013 to 2020 (screening from 2015). Targeted screening was implemented in 2015 for 2 nurseries, from 2016 to all nurseries	Infants failing NHS	Primary PCR on saliva	8 confirmed/528 tested for CMV/891 failing NHS	n.a.	6	valganciclovir	hearing stable in 3, progressed in 2, improved in 1.	Hearing function improved in 1 patient out of 6 diagnosed and treated for isolated SNHL. Not specified if symptomatic infants were excluded from the study.
Khi Chung, 2022 [66] Netherlands	National, prospective observational From 2012 to 2016	Infants failing NHS (three rounds: two OAEs, one ABR)	PCR on DBS	54 confirmed/1374 DBS screened/1381 infants failing NHS	n.a.	n.a. (48 infants had confirmed HL, but other concurrent symptoms were not excluded or specified in the study)	n.a	n.a.	Symptomatic children were not excluded and granular data about clinical scenario were not provided.
Fourgeaud, 2022 [67] France	Multicenter, prospective study From 2014 to 2017	Newborns failing NHS (twice OAE in 3 centers, twice ABR in 2 centers)	PCR on saliva Confirmatory test on saliva and blood	2 confirmed/231 screened for CMV/236 failing NHS	n.a.	n.a. (2 cases of HL but no information on other symptoms)	valganciclovir	n.a.	No granular data about clinical scenario of confirmed cases. Not specified if symptomatic infants were excluded from the study.
Webb, 2022 [68] Australia	Prospective, multicenter From June 2019 to March 2020	Infants failing NHS	PCR on saliva, confirmed on urine and plasma	1 positive out of 96 tested	0	1	valganciclovir started at 32 days of life for 6 months	n.a.	Good feasibility and acceptability.
Zhang, 2023 [69] Japan	Single-center observational prospective study From October 2018 to October 2021	Newborns with suggestive perinatal conditions, including failing NHS (twice ABR)	PCR on urine	1 positive out of 12 failing NHS, 1 positive screened because of abnormal CNS findings, 1 positive screened for suspected maternal infection during pregnancy	n.a.	1	2 treated with valgancyclovir	n.a.	No clinical outcome.

### 3.4. Vaccinations

Key factors considered in the vaccine prioritization strategies include disease burden, vaccine effectiveness and safety, the feasibility of additional recommendations in the context of the existing vaccination schedule, equity of access, and whether vaccination is a good use of public funds [70].

Cytomegalovirus is not highly contagious, and it is believed that protecting 50–60% of the population could lead to viral elimination, making vaccination cost effective [71]. Several efforts are currently focused on vaccine development, with some aimed at preventing infection in transplant patients and others targeting the prevention of congenital infection [72].

Regarding cCMV disease, vaccination efforts should focus on protecting women of childbearing age from acquiring the infection. To achieve this goal, the target population should primarily include young women, adolescents, and young children as they are the main sources of infection for pregnant mothers [73]. 

Here is a summary of vaccines studied in the population of interest, which includes adolescents, children, and women of childbearing age (Table 3). Studies on transplant recipients and phase 1 trials are excluded from this summary.

Three CMV vaccines have reached phases 2 and 3 in healthy individuals. The MF59-adjuvated recombinant CMV glycoprotein B vaccine was studied in two phase 2 trials in post-partum seronegative women and adolescents [74,75]. While in the first study, the vaccine’s effectiveness in preventing the infection in post-partum women seemed good [74], in the second study on adolescents, it was not significant. No phase 3 trial is ongoing. V160 is a whole-virus vaccine derived from the live-attenuated AD169 strain. A recently published phase 2 trial by Das was terminated early because of the futility in preventing the primary CMV infection in vaccinated women compared to the placebo [76]. The mRNA-1647 vaccine seem to induce higher neutralization and antibody-dependent cellular cytotoxicity responses compared to the gB/MF59 vaccine [77]. A phase 2 trial of the CMV mRNA-1647 vaccine was terminated in 2023 [78]. The results are not available, but in 2020, the company declared a positive seven-month interim safety and immunogenicity analysis. The following phase 3 trial is expected to evaluate the efficacy of the vaccine in a larger population of healthy individuals at risk of having early post-vaccination contact with young children (NCT05085366) [79]. Moreover, 7545 patients were enrolled, but the results have still not been reported.

The immunogenicity of CMV vaccines has not been studied in young children. This is due to the lack of positive effectiveness data on adults, unknown duration of immunity, and mildness of the disease in infancy [73].

**Table 3:** Summary of studies (published or ongoing) included on vaccination for CMV. Nab: neutralizing antibody.

Vaccine	Author, Year or Trial ID Number	Study Design	Population	Outcome	Enrolment Time	Results
bB-MF59: MF59 adjuvated recombinant CMV envelope glicoproteinB subunit	Pass, 2009 [74]	Phase 2, placebo-controlled, randomized, double-blind trial.	Post-partum, seronegative women, aged 14–40 years and healthy.	Effectiveness in preventing CMV infection during a 42-month period	August 1999 to April 2006	464 subject enrolled. Vaccine recipients were more likely to remain uninfected than placebo recipients (*p* = 0.02).
bB-MF59: MF59 adjuvated recombinant CMV envelope glicoproteinB subunit	Bernstein, 2017 [75]	Phase 2, multicenter, randomized, double-blind, controlled study.	Healthy adolescent females.	Effectiveness in preventing CMV infection, immunogenicity, safety.	June 2006–June 2013	402 subjects enrolled. CMV infection occurred without significant differences between vaccinated and control individuals.
V160: whole-virus vaccine that is derived from the live-attenuated AD169 strain	Das, 2023 [76]	Phase 2b, multicenter, randomized, double-blind, placebo-controlled study.	Healthy, CMV-seronegative, non-pregnant, 16–35-year-old women of childbearing potential with exposure to children aged 5 years or younger.	Efficacy of three doses of V160 in reducing the incidence of primary CMV infection during the follow-up period starting 30 days after the last dose of vaccine; vaccine safety.	April 2018–August 2019	2220 enrolled. The vaccine efficacy for the V160 three-dose group was 44.6% (95% CI −15.2 to 74.8) at the final testing of the primary efficacy hypothesis, a result corresponding to failure to demonstrate the primary efficacy hypothesis. The study was terminated due to futility.
mRNA-1647	NCT04232280 [78]	Phase 2, randomized, observer-blind, placebo-controlled, dose-finding trial. Part 1: to inform the selection of the middle dose level for further development. Part 2: to further evaluate the safety and immunogenicity of the middle dose level of mRNA-1647 vaccine or placebo.	Healthy participants seropositive or seronegative, males or females, 18 to 40 years of age.	Safety, immunogenicity (NAb titers)	September 2020–April 2023	315 subjects enrolled. No results reported.
mRNA-1647	Ongoing trial NCT05085366 [79]	Phase 3, randomized, observer-blind, placebo-controlled study.	Participants aged ≥20 years, has or anticipates having direct exposure within 7 months after the planned first dose (in the home, socially, or occupationally) to at least 1 child ≤5 years of age. Enrollment estimated 6900 subjects.	Efficacy (seroconversion from a negative to a positive result) in females and in all participants. Safety.	October 2021–April 2024	Enrolled 7454 patients, no results reported.

## 4. Final Considerations and Future Prospectives

Despite being a widespread disease with a substantial body of literature addressing congenital CMV, evidence regarding the effectiveness of various interventions remains weak. The absence of effective treatment options for asymptomatic children or LO-SNHL complicates the implementation of screening by health systems. This underscores the need, at higher levels, to weigh the allocation of public funds against numerous competing health priorities. The promotion of trials during pregnancy is primarily hindered by ethical concerns, which limits the advancement of therapeutic strategies to prevent fetus damages by CMV infection. It is crucial to enhance education among pregnant women and increase awareness among healthcare providers about congenital CMV at the community, local, and national organizational levels. Early education remains, in fact, the main useful strategy to prevent primary maternal infection during pregnancy, and awareness initiatives should be supported by health systems.

To date, vaccine research has predominantly focused on immunosuppressed patients, and data regarding healthy women do not demonstrate significant effectiveness. The network between gynecologists, obstetricians, infectious disease specialists and pediatric infectious disease teams for managing mother–infant pairs should be strengthened and established as the standard of care for cCMV by public health systems. In this view, maternal and neonatal universal screening emerges as a possible way forward, along with continued research on vaccines. It would ease the understanding of risk factors for infection and sequelae (such as prematurity, low birth weight, and brain imaging anomalies), the development of scoring systems, and the optimization of follow-up modalities. This would allow researchers to construct a basis for new therapeutic trials.

## Data Availability

All data are included in the manuscript.
